# Supplementary material for: Utility of surveillance data for planning for dengue elimination in Yogyakarta, Indonesia: a scenario-tree modelling approach
Source: BMJ Glob Health. 2023 Nov 20;8(11):e013313. doi: 10.1136/bmjgh-2023-013313 (PMC10660636; doi:10.1136/bmjgh-2023-013313)

Supplementary information

Supplementary Table 1: Summary description of multiple separate simulations conducted

Simulation	Change from default simulation	New distribution parameters	Median system sensitivity (95% PI)*
1	Default parameters as described	No change	0.131 (0.111 – 0.152)
2	Probability of seeking healthcare at puskesmas participating in the enhanced dengue surveillance program reduced proportionally by 20%, reflecting a decrease in the number of puskesmas participating in enhanced surveillance due to resource constraints	pert(0.271, 0.339, 0.407)	0.105 (0.089 – 0.122)
3	Probability of first seeking healthcare at hospital (i.e., without first presenting at puskesmas participating in the enhanced dengue surveillance) increased by 5% (absolute)	pert(0.136, 0.194, 0.223)	0.132 (0.112 – 0.153)
4	Probability of sampling at puskesmas participating in the enhanced dengue surveillance program increased, reflecting increased awareness of the dengue elimination goal amongst healthcare providers	pert(0.8, 0.9, 0.95)	0.142 (0.121 – 0.163)
5	Probability of sampling at hospital increased, reflecting increased awareness of the dengue elimination goal amongst healthcare providers	pert(0.7, 0.8, 0.9)	0.138 (0.118 – 0.159)
6	Probability of notification of a positive result at a hospital increased, reflecting increased awareness of the dengue elimination goal amongst healthcare providers	pert(0.9, 0.95, 0.98)	0.130 (0.110 – 0.151)
7	Changes per simulations 3 – 6 combined in a single model		0.162 (0.142 – 0.184)

Table notes: *Assumes a probability of recurrence of local dengue transmission of 1% per month.

Bannister-Tyrrell M, *et al. BMJ Glob Health* 2023; 8:e013313. doi: 10.1136/bmjgh-2023-013313

Supplementary Table 2: Unit sensitivities (USe) by age, gender, and type of health facility

	Median USe (5th-95th percentile)	
Risk stratum	Puskesmas	Hospital
Under 5_female	0.138 (0.118-0.159)	0.016 (0.012-0.022)
5 to 14_female	0.123 (0.105-0.141)	0.015 (0.011-0.02)
15 to 29_female	0.108 (0.093-0.125)	0.013 (0.009-0.017)
30 and over_female	0.108 (0.093-0.125)	0.013 (0.009-0.017)
Under 5_male	0.138 (0.118-0.159)	0.016 (0.012-0.022)
5 to 14_male	0.123 (0.105-0.141)	0.015 (0.011-0.02)
15 to 29_male	0.108 (0.093-0.125)	0.013 (0.009-0.017)
30 and over_male	0.108 (0.093-0.125)	0.013 (0.009-0.017)

Supplementary Figure 1: Median confidence of elimination over 60 months for seven alternative simulations and assuming a probability of recurrence of 1% per month.

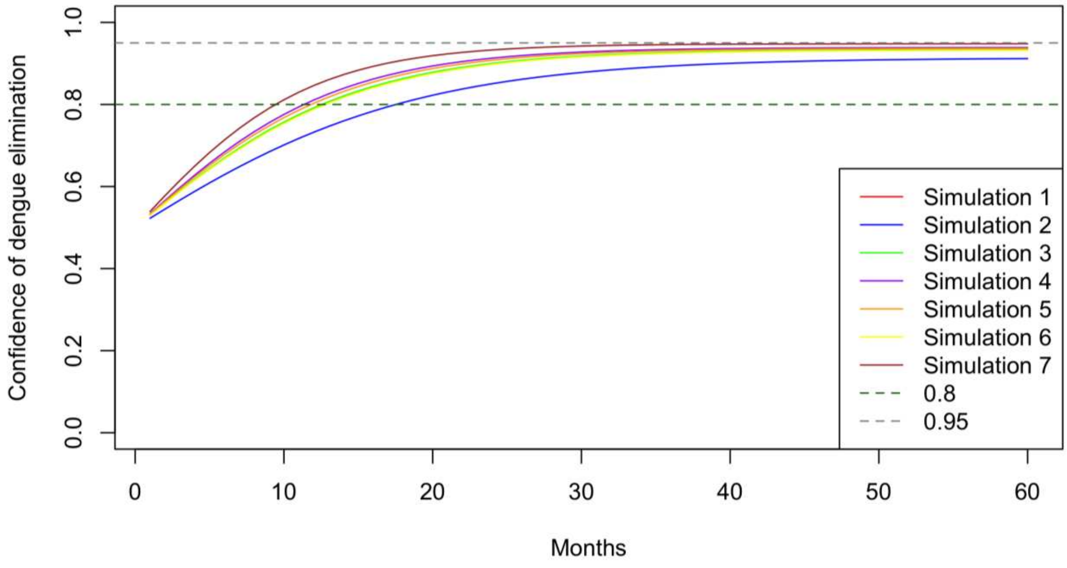

Supplement: Supplementary data [file bmjgh-2023-013313supp001.pdf]
